# A Large‐Scale Cross‐Population Evaluation of Genetic Risk Variants for Dilated Cardiomyopathy in Dobermanns

**DOI:** 10.1002/age.70206

**Published:** 2026-09-09

**Authors:** Alma H. Hulsman, Gwen Flokstra, Christina Kijan, Marilijn van Rumpt, Johannes C. M. Vernooij, Thomas C. Nelson, Sophie Liu, Lieke Vree, Magdalena Harakalova, Ingrid Ljungvall, Jens Haggstrom, Gerhard Wess, Hille Fieten, Marjo K. Hytönen, Hannes Lohi, Frank G. van Steenbeek

**Affiliations:** ^1^ Expertise Centre for Veterinary Genetics, Department Clinical Sciences, Faculty of Veterinary Medicine Utrecht University Utrecht the Netherlands; ^2^ Department Population Health Sciences, Faculty of Veterinary Medicine Utrecht University Utrecht the Netherlands; ^3^ Embark Veterinary, Inc. Boston Massachusetts USA; ^4^ Doberman Diversity Project Oakland California USA; ^5^ Department of Cardiology, Division Heart & Lungs University Medical Center Utrecht (UMCU) Utrecht the Netherlands; ^6^ Department of Clinical Sciences Swedish University of Agricultural Sciences Uppsala Sweden; ^7^ LMU Small Animal Clinic, Ludwig Maximilians University of Munich Munich Germany; ^8^ Department of Veterinary Biosciences, Faculty of Veterinary Medicine University of Helsinki Helsinki Finland; ^9^ Department of Clinical and Medical Genetics, Faculty of Medicine University of Helsinki Helsinki Finland; ^10^ Folkhälsan Research Center Helsinki Finland

**Keywords:** canine, genotype imputation, population structure, risk prediction, RNF207

## Abstract

Previous studies suggest that genetic risk factors for dilated cardiomyopathy (DCM) in Dobermann dogs may differ between populations, complicating the use of genetic tests across subpopulations. Variants in pyruvate dehydrogenase kinase 4 (*PDK4*) and titin (*TTN*) have been associated with DCM in North American Dobermanns, whereas two loci on chromosome 5 involving Protein Kinase AMP‐Activated Catalytic Subunit Alpha 2 (*PRKAA2*) and Ring Finger Protein 207 (*RNF207*) have been implicated in European populations. To assess the consistency of these signals across populations of Dobermanns, we harmonized and imputed genotype data from a Discovery cohort (*n* = 444) and a Replication cohort (*n* = 1771), and supplemented these with allele frequency data from a large diversity cohort (*n* = 3226) for cross‐platform analyses. We detected a shared association signal near *RNF207* in affected dogs in both Europe and North America. In contrast, SNPs located in and near *PDK4* and *TTN* variants showed no association with DCM in either population. These findings indicate that the *RNF207* variant represents a shared genetic risk factor for DCM in Dobermanns across populations, and support the dog, particularly the Dobermann, as a relevant spontaneous model for RNF207‐driven DCM. The previously reported associations between *PDK4* and *TTN* variants and DCM were not replicated in the North American Dobermann population, nor in the European Dobermann populations. Also, these results highlight the need for population‐specific validation and functional validation of identified variants where possible, before implementation of variants in genetic testing strategies.

## 1 Introduction

Dilated cardiomyopathy (DCM) is the second most common cardiac disease in dogs (Tidholm et al. 2001; Egenvall et al. 2006; Lewis et al. 2018; Stern and Ueda 2019). During the preclinical stage, affected dogs may exhibit either morphological and functional abnormalities detectable by echocardiography or electrical abnormalities identified by standard and/or 24‐h (Holter)electrocardiography. Affected dogs may experience sudden cardiac death or develop congestive heart failure (CHF) (Tidholm et al. 2001). Certain breeds, such as the Dobermann, are overrepresented, suggesting a strong genetic influence. A European study (Wess et al. 2010) reported a cumulative prevalence of DCM in Dobermanns of 58.2%. The prevalence increased with age, ranging from 3.3% in dogs younger than 2 years, 9.9% in dogs aged 2–4 years, 12.5% in dogs aged 4–6 years, 43.6% in dogs aged 6–8 years, and 44.1% in dogs older than 8 years. The Dobermann originated in Germany in the 1890s and was exported to North America in the early 1900s. Subsequent geographic separation and differing breeding practices focusing on either show or working lines have led to genetic differentiation into distinct subpopulations within the breed (Lampi et al. 2020; Wade et al. 2023).

In North America, reported genetic variants associated with DCM in Dobermanns include mutations in the pyruvate dehydrogenase kinase 4 (*PDK4*) and titin (*TTN*) genes. The *PDK4* variant consists of a 16–base pair deletion affecting the 5′ donor splice site of intron 10 and is inherited in an autosomal dominant manner, with a reported penetrance of 68% in the studied population (Meurs et al. 2012). The *TTN* variant is a single base pair substitution (C>T) that also shows autosomal dominant inheritance, with an estimated penetrance of 47% (Meurs et al. 2019; Shen et al. 2022). A subsequent North American study of 48 Dobermanns with DCM found that 28 dogs carried the *TTN* variant, four carried the *PDK4* variant, 10 carried both variants, and six carried neither (Meurs et al. 2020). However, these *PDK4* and *TTN* variants were not associated with DCM in European Dobermann populations (Owczarek‐Lipska et al. 2013; Niskanen et al. 2023; Dutton et al. 2025).

In Europe, a DCM‐associated locus on chromosome 5 was identified in Dobermanns, linked to left ventricular systolic dysfunction and ventricular premature complexes (Mausberg et al. 2011). This locus was later confirmed and refined, leading to the identification of a variant in the Ring Finger Protein 207 (*RNF207*) gene and a deletion affecting the mRNA sequence of Protein Kinase AMP‐Activated Catalytic Subunit Alpha 2 (*PRKAA2*) in a predominantly central European Dobermann population (Niskanen et al. 2023). The *RNF207* variant was functionally validated at both the RNA and protein levels, supporting its potential role in DCM pathogenesis. This variant was subsequently found to be associated with DCM in Dobermanns in the United Kingdom (Dutton et al. 2025).

The current screening guidelines for DCM in Dobermanns, published by the European Society of Veterinary Cardiology in 2017, are based on clinical evaluation using echocardiography and 24‐h Holter electrocardiography (Wess et al. 2017). Although these guidelines do not include recommendations for genetic testing, breeders frequently use commercially available genetic tests to inform breeding decisions. Commercially available genetic tests for DCM in Dobermanns include DCM1 (*PDK4*) and DCM2 (*TTN*), and since 2023, have been expanded to include DCM3 (*PRKAA2*) and DCM4 (*RNF207*). However, because not all these tests are relevant to all Dobermann subpopulations, their use in breeding strategies may unintentionally contribute to a further reduction in the genetic diversity of the breed.

This study aimed to validate DCM risk alleles (DCM3 and DCM4) in a North American subpopulation and to assess their relevance for breed health and breeding strategies. Specifically, we identified and evaluated genetic risk alleles associated with DCM, assessed the strength of their association with the disease phenotype, and determined their predictive value across genotypic profiles. In addition, genetic differences between European and North American subpopulations and the prevalence of DCM3 and DCM4 in North American Dobermanns were examined.

## 2 Materials and Methods

### 2.1 Study Cohorts

To ensure broad representation of the Dobermann breed, we included datasets from different genotyping platforms, covering a total of 5087 Dobermanns.

Discovery cohort: In our study, previously published genotype data from 444 Dobermanns (Niskanen et al. 2023), which were used for the discovery of DCM3 and DCM4, were included. These samples were genotyped using the Axiom Canine Genotyping Array Sets A and B, covering 1 269 218 markers. After initial quality‐control filtering, the dataset contained 859 223 SNPs and included cases from the “echo only,” (dogs with an abnormal echocardiography result, a normal Holter result, and no CHF), “echo + arrhythmia,” (dogs with an abnormal result for both echocardiographic examination and 24‐h Holter monitoring and no CHF), “CHF,” (dogs at the clinical stage of DCM) and “Utrecht” (clinically affected dogs and controls older than 9 years) cohorts. Detailed descriptions of these cohorts and the corresponding case/control definitions are provided in the original study report.

Replication cohort: Genotype and phenotype data for 1771 Dobermanns were obtained through Embark Veterinary Inc., which collected DNA samples for genetic screening and health assessment with owner consent. Owners also completed a Qualtrics questionnaire reporting their dogs' health status, which was used for phenotyping. Questionnaire responses were manually reviewed for inconsistencies and incomplete information. The list of questionnaire items is provided in the Supporting Information. Only questionnaire items relevant to the objectives of the present study are presented here. Dogs were classified as controls if they were over 9 years old with no clinical signs suggestive of DCM, or if they were older than 8 years and had normal annual echocardiographic and Holter evaluations. Cases were defined as dogs whose owners reported that a veterinarian had diagnosed DCM based on echocardiography with or without 24‐h Holter monitoring. All dogs had been genotyped previously using a custom Illumina CanineHD SNP array (229 991 SNPs).

Allele frequency cohort: Additionally, genotype data from the Doberman Diversity Project (DDP) were obtained, which included 3226 Dobermanns with unknown health status (Polenberg et al. 2024). These dogs had been genotyped by Embark using a custom Illumina CanineHD SNP array (216 184 SNPs), and the data were collected and shared with the DDP with owner consent.

### 2.2 Data Harmonization

Because the genotype datasets originate from different SNP array platforms, harmonization was required before imputation. Genotype data were therefore processed using a harmonization pipeline developed at the Expertise Centre for Veterinary Genetics, Utrecht University (https://github.com/UtrechtUniversity/Canine_SNP_Harmonize), which enables the merging of datasets generated on multiple genotyping platforms. Raw SNP data were converted into a uniform format (TOP strand calling) using platform‐specific scripts developed in Python 3.12.3 and PLINK 2.0 (Chang et al. 2015). Harmonization is performed across several aspects, including differences in SNP identifiers, indel notation, genome build and genomic coordinates, allele coding, sex chromosome notation, and pseudo‐autosomal region definition. All SNP datasets were based on the CanFam3.1 reference genome (GCF_000002285.3).

### 2.3 Quality Control

Quality control (QC) steps were applied to all genotype datasets before imputation. A second automated in‐house pipeline (https://github.com/UtrechtUniversity/Canine_SNP_Quality_Control) was used to perform sample‐level QC, including sample call rate check, sex determination, duplicate sample detection, and breed verification. Further SNP‐level QC was conducted separately.

Samples with a call rate below 90% were excluded. For sex determination, platform‐specific approaches were used. For Embark array data (Replication and Allele frequency cohort), sex was inferred based on the number of reliably called Y‐chromosomal SNPs. Prior to this analysis, Y‐SNPs that were frequently called in female samples were removed to avoid false assignments. Samples with more than 100 remaining Y‐SNPs were classified as male. For the Axiom dataset (Discovery cohort), which does not include Y‐chromosomal markers, sex was inferred based on X‐chromosomal homozygosity: samples with X‐chromosome homozygosity above 98.5% were classified as male, while samples below 97% were classified as female.

Duplicate samples were identified (kinship score > 0.4) using the KING‐robust kinship estimator (Manichaikul et al. 2010) implemented in PLINK 2.0, and for each duplicate pair, the sample with the lowest SNP call count was removed. Breed verification was performed using the *neighbor* program from the PHYLIP package. A reference phylogenetic tree was constructed using genotype data from 289 dog breeds, each represented by five individuals. Study samples were added to this reference tree, and breed identity was assessed based on clustering within the expected breed‐specific clade. Samples clustering outside the Dobermann clade or showing evidence of admixture with other breeds were excluded.

Further SNP‐level QC was conducted using PLINK 1.9. Variants with a call rate below 97% (‐geno 0.03) were removed. SNPs with a minor allele frequency (MAF) below 1% (‐maf 0.01) or deviating from Hardy–Weinberg equilibrium (HWE *p* < 5 × 10^−5^; ‐hwe 0.00005) were excluded. Heterozygous genotype calls at X‐linked SNPs in males were recoded as missing data.

### 2.4 Genotype Imputation

To increase SNP density in the target datasets (Replication and Allele frequency cohort), genotype imputation was performed to infer missing genotypes using haplotype information from the Axiom SNP array, which was used as the reference dataset. To prepare datasets for imputation, genotypes were converted to forward‐strand orientation, set to diploid using the *fixploidy* plugin in BCFTools, and Y‐chromosomal and mitochondrial SNPs were removed, as these are not included in the Axiom reference panel. Imputation followed a two‐step procedure. First, the Axiom Discovery cohort dataset was phased to obtain high‐quality haplotypes. These phased haplotypes were subsequently used as a reference panel for both phasing and imputation of the target datasets. Phasing and imputation were carried out for the Replication and Allele frequency cohort separately, and separately for each autosomal chromosome using a Hidden Markov Model (HMM) framework as implemented in Beagle v5.5 (Browning et al. 2018, 2021). An effective population size parameter of Ne = 200 was used, based on estimates reported for Dobermann populations (Wade et al. 2023). All other parameters were kept at their default values. Imputation accuracy was assessed by Beagle's estimated squared correlation between imputed and true genotypes (DR2), and by calculating genotype concordance. Four samples genotyped both on the target and reference array, three from the Allele frequency cohort and one from the Replication cohort, were excluded from the phased Axiom Discovery cohort reference panel prior to imputation and subsequently used for checking concordance, by comparing imputed genotypes against their original array‐based genotypes. In addition, one sample from the Replication cohort for which whole‐genome sequencing data were available was included to assess concordance between imputed and sequence‐derived genotypes. Following imputation, both target and reference datasets were merged, retaining only variants with a SNP call rate of at least 95% across all datasets.

### 2.5 Population‐Specific Association Analysis

To explore the population structure of the merged phenotype‐linked dataset, a principal component analysis (PCA) was conducted in PLINK 1.9. The PCA was based on a variance‐standardized relationship matrix. The geographic origin of the samples was defined based on the two clusters in the PCA plot. Genome‐wide association studies (GWAS) were conducted using GEMMA 0.98.5 (Zhou and Stephens 2012), a software package that employs a linear mixed model approach to account for population stratification and relatedness. A minor allele frequency threshold of 0.01 was applied to filter out rare variants. Furthermore, a Bonferroni correction was used to adjust for multiple testing. Allele frequencies of SNPs of interest were calculated using the freq flag in PLINK 1.9. The association results were visualized in R 4.4.3 using the packages “qqman”, “ggplot2”, and “dplyr”.

### 2.6 Risk Prediction

A logistic regression model with binomial distribution was used with phenotype for DCM as the outcome variable and factors sex and DCM3 and DCM4 allele frequency as explanatory variables. For this analysis, all Dobermanns that met the case/control definitions originating from the Discovery and Replication cohort were used. The results are presented as Odds ratios with 95% confidence interval. Secondly, cross‐validation was applied to predict the risk for DCM in a new population. The full data were split into a train set with randomly drawn 180 cases and 180 controls (65.7%), and the remaining dogs (148 cases, 40 controls; 34.3%) were used as a test set. We assume that the dogs in the train set are independent from the dogs in the test set. The above‐described model was applied to the training set, and the model estimates were used to predict the risk for DCM for the dogs in the test set. The training and test approach was applied 100 times with a new random set of samples for the training and test set, and from each run, the model estimates and the predicted probability for DCM of the dogs in the test set were saved for further evaluation. The predicted probabilities and true DCM status of the dogs were studied in a Receiver Operation curve analysis (R package pROC (Robin et al. 2011)). The mean predicted probability per dog from the test set was used as predictor, and the phenotype was used as the response. Sensitivity and specificity were calculated and reported. The model estimates of the 100 runs were used to predict the risk probability of 18 genotypic profiles defined by sex (F, M), DCM3 risk alleles (0, 1, 2), and DCM4 risk alleles (0, 1, 2). The predicted probabilities were visualized by boxplots showing all 100 predictions for each of the genotypic profiles.

## 3 Results

### 3.1 Data Cleaning and Imputation

Due to overlapping sample identities both across and within datasets, particularly between the Replication and Allele frequency cohorts, 354 duplicate samples were excluded. Four samples from the Replication cohort were excluded due to a sample call rate below 90%, and one sample of the Replication cohort failed to meet breed verification criteria. After initial preprocessing of the genotype data using the quality‐control steps, the number of SNPs remaining is shown in Table 1. Using the phased Discovery cohort Axiom dataset as the reference panel, imputation resulted in 487 652 SNPs for the Replication cohort and 479 321 SNPs for the Allele frequency cohort. The three datasets were merged with a 95% SNP call rate threshold, yielding a final dataset of 462 386 SNPs in a total of 5078 Dobermanns (Table 1, CONSORT‐style sample flow diagram as Figure S1).

**TABLE 1:** Number of SNPs and samples per dataset before and after quality control and imputation.

Cohort	Type	Initial SNPs	SNPs after quality control	SNPs after imputation	Samples after quality control
Discovery cohort	Reference	859 223	462 386	—	440
Replication cohort	Target	229 991	102 910	487 652	1766
Allele frequency cohort	Target	216 184	77 563	479 321	2872
Full dataset (imputed targets + reference)	—	—	—	462 386	5078

*Note:* The mean imputation accuracy (DR2) of all imputed SNPs (based on the whole imputed dataset) was 0.97. The concordance for each of the five samples tested exceeded 99.4%, regardless of the *R*‐squared value.

### 3.2 Combined Dataset of Discovery and Replication Cohort

A total of 548 dogs met the case/control definitions, including 378 dogs from the Discovery cohort (total 440) and 170 dogs from the Replication cohort (total 1766). Most of the excluded dogs from the Replication cohort either lacked phenotype data or did not meet the age threshold of over 8 years required for controls. Excluded dogs from the Discovery dataset had missing phenotype or genotype data. These excluded dogs from both datasets were pooled with the Allele frequency cohort. In total, this large Allele frequency cohort consisted of 4530 Dobermanns.

**FIGURE 1:**
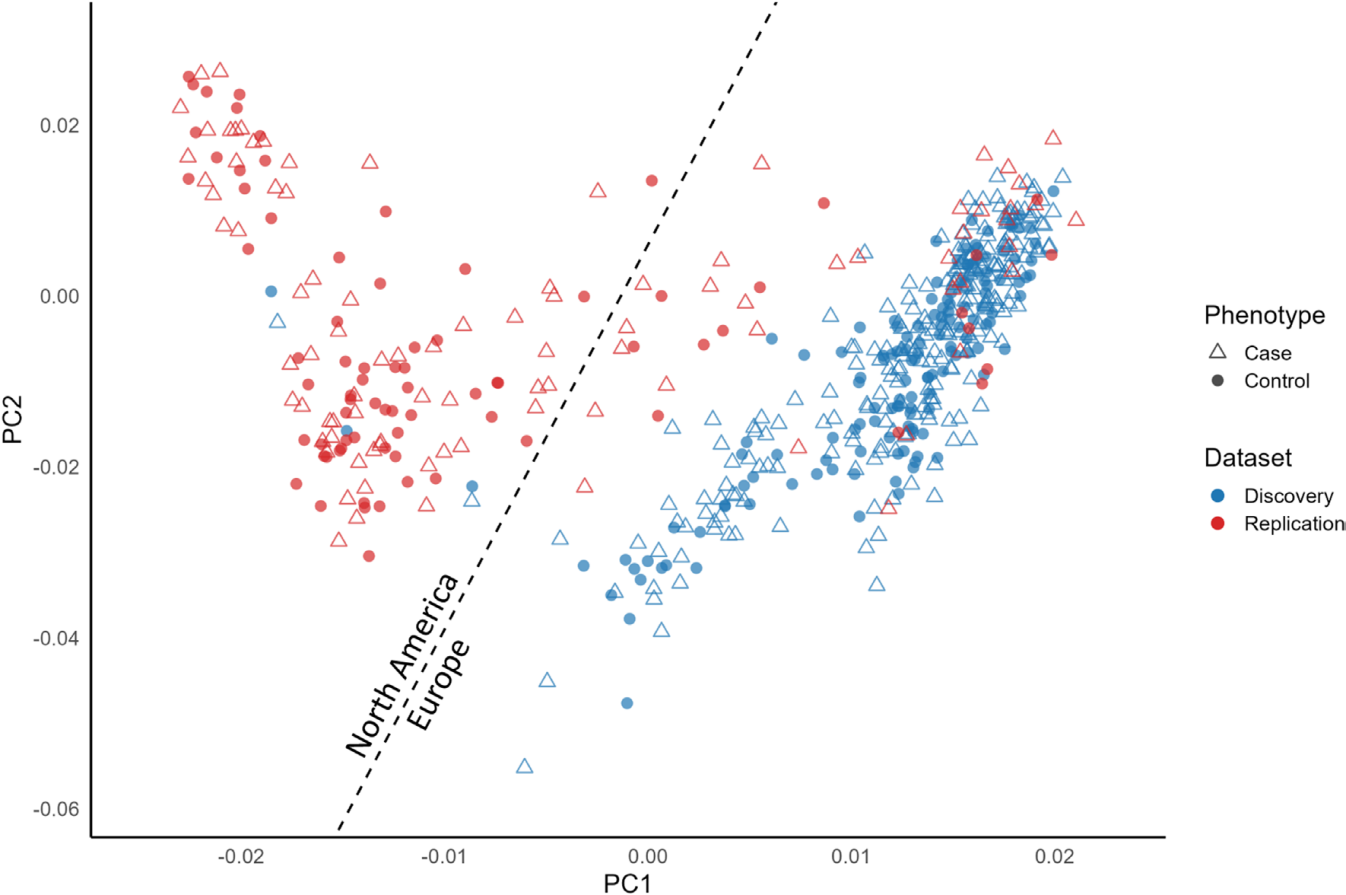
Principal component analysis based on the combined Discovery and Replication dataset. Each point represents an individual, colored by dataset and geographic origin. The dashed line marks the approximate boundary between the North American (*n* = 127) and European (*n* = 421) populations.

To assess the genetic structure of the phenotype‐linked merged Discovery and Replication datasets (*n* = 548), a PCA was performed. The first two principal components revealed two distinct genetic clusters, corresponding to a European and a North American sub‐population. The observed population division closely reflected the distinction between the original Discovery and Replication datasets, which predominantly consisted of dogs originating from Europe and North America, respectively. K‐means clustering was performed to correct for the population‐driven clusters (Figure 1). After correction, the European population consisted of 421 dogs (comprising 266 cases and 155 controls), and the North American population consisted of 127 dogs (comprising 62 cases and 65 controls).

### 3.3 GWAS of the Combined Dataset

**FIGURE 2:**
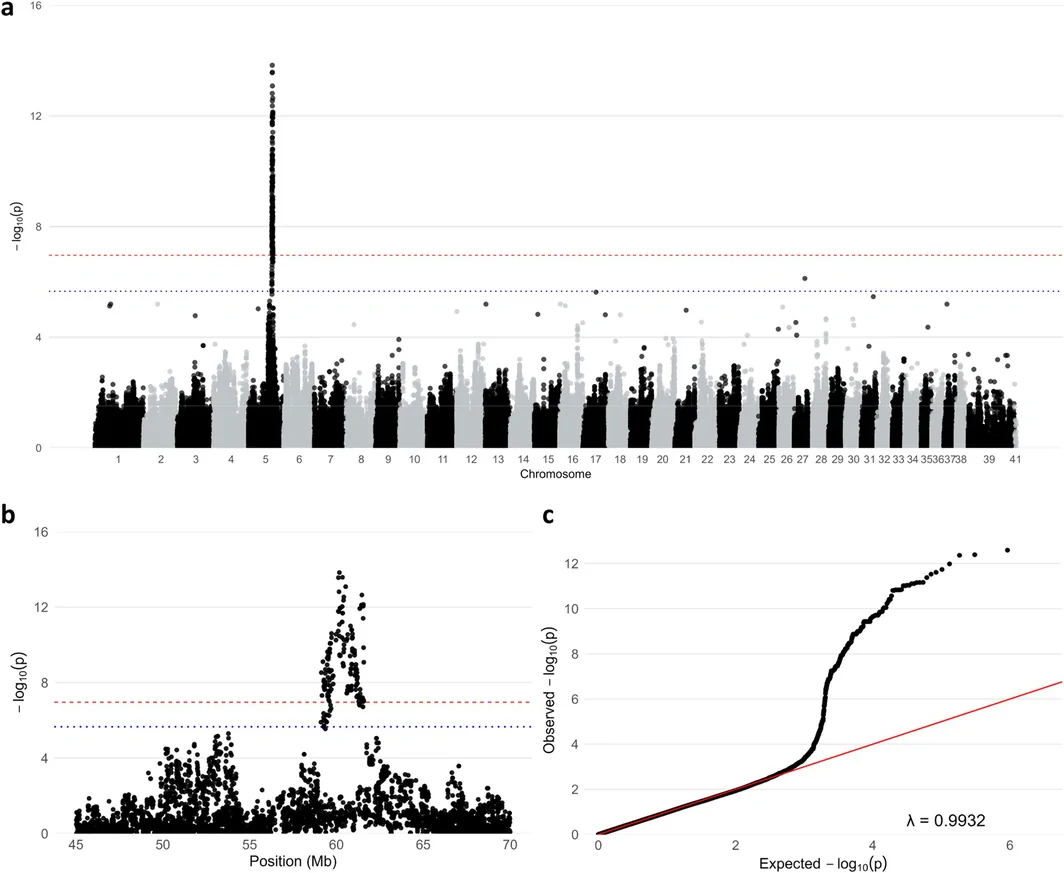
GWAS results of the combined Discovery and Replication dataset. Results of a univariate linear mixed model analysis including 548 dogs and 458 058 SNPs. (a) Manhattan plot showing the association between genetic variants and DCM. The red dashed line marks the Bonferroni‐corrected significance threshold; the blue dotted line marks the suggestive threshold. (b) A locus plot of chr5:45–70 Mb. (c) QQ‐plot of the *p*‐values.

To identify loci associated with DCM, a GWAS on the combined Discovery and Replication datasets was performed, comprising a total of 548 dogs (328 cases and 220 controls). After additional quality control in GEMMA, 458058 SNPs were included in the analysis. Figure 2 provides an overview of the GWAS results. Consistent with previous findings in the Discovery cohort (top SNP at position chr5:60531090 in the major locus (Niskanen et al. 2023)), the strongest association signal was observed at chromosome 5. Our analysis identified a top SNP at chr5:60168651 (*p*_*wald*_ = 1.46 × 10^−14^), located closer to the *RNF207* variant (chr5:60111983) than the previously reported top SNP (chr5:60531090).

A suggestive peak at chromosome 5 around 53 Mb was observed, which corresponds to previous results obtained from the Discovery dataset. However, in our analysis, this second peak did not reach genome‐wide significance (Figure 2b).

### 3.4 North American Dataset

**FIGURE 3:**
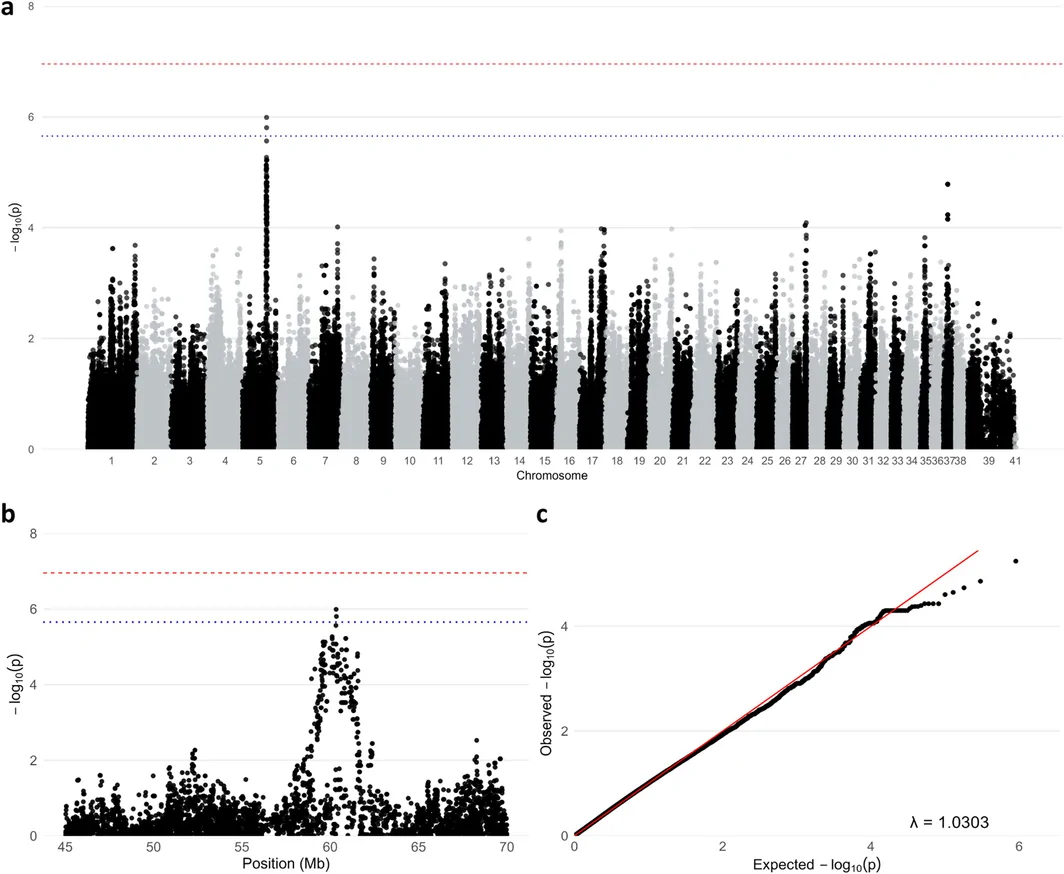
GWAS results of the North American cohort. Results of a univariate linear mixed model analysis including 127 dogs and 450 833 SNPs. (a) Manhattan plot showing the association between genetic variants and DCM. The red dashed line marks the Bonferroni‐corrected significance threshold; the blue dotted line marks the suggestive threshold. (DCM3 chr5:53109178; DCM4 chr5:60111983) (b) A locus plot of chr5:45–70 Mb. (c) QQ‐plot of the *p*‐values.

We next examined whether the signal at chromosome 5 remained evident when restricting the GWAS to the 127 dogs in our North American cohort, which was primarily derived from the Replication cohort. This North American population included 62 cases and 65 controls (36 males, 29 females). The cases included 34 males (average age 5.9 years) and 28 females (average age 7.2 years). The final dataset comprised 450 833 SNPs after quality control in GEMMA. A strong signal on chromosome 5 was identified (Figure 3), with the most prominent peak around 60 Mb. The strongest association signal in this peak was at 5:60332726 (*p*_*wald*_ = 1.02 × 10^−6^). The minor peak around 53 Mb was lost in the North American cohort (Figure 3b).

**FIGURE 4:**
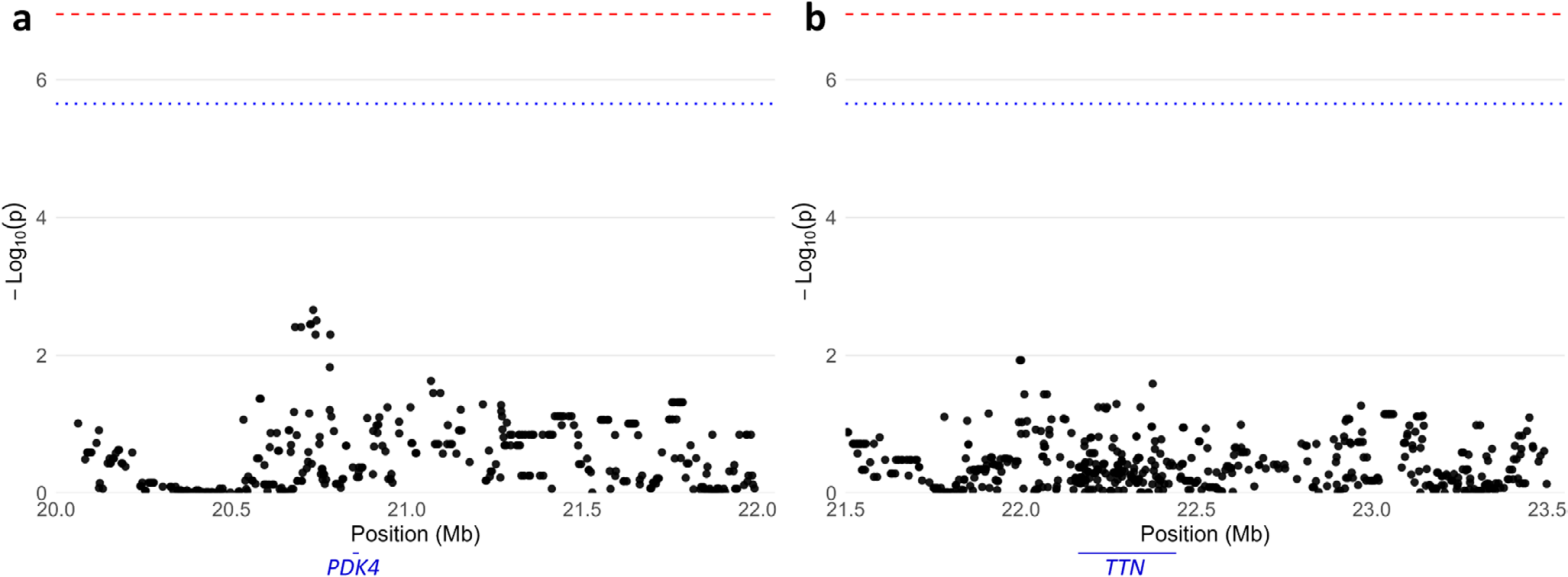
SNP regions near *PDK4* (DCM 1) and *TTN* (DCM 2). Results are shown from the GWAS of the North American population. (a) A locus plot of chr14:20.0–22.0 Mb. The blue horizontal line beneath the *x*‐axis indicates the location of the PDK4 variant. (b) A locus plot of chr36:21.5–23.5 Mb. The blue horizontal line beneath the *x*‐axis indicates the location of the *TTN* variant.

To assess the screening potential for DCM1 and DCM2 in the North American population, the SNP regions located near *PDK4* and *TTN*, respectively, were assessed. The allelic association did not reveal suggestive or significant association signals at these SNP regions (Figure 4). However, *PDK4* and *TTN* variants were not directly genotyped.

To further assess the prevalence of the identified DCM4 risk allele, the allele frequencies of the top SNP at position chr5:60168651 in the Allele frequency cohort were determined. Of the 4530 Dobermanns with unknown phenotypes, 2262 dogs clustered within the North American population (see PCA plot in Figure S2). In the North American population within the Allele frequency cohort, the top SNP at the major locus presented an allele frequency of 0.53 for the associated risk allele T (Table 2).

**TABLE 2:** Prevalence of genotypes and allele frequencies for the risk alleles of DCM3 and DCM4 in the different study populations. 0 = homozygous reference; 1 = heterozygous; 2 = homozygous variant.

cohort	Status	DCM3 (Top SNP chr5:53062339)				DCM4 (Top SNP chr5:60168651)			
		0 (AA)	1 (AG)	2 (GG)	Risk allele (G) Freq	0 (AA)	1 (AT)	2 (TT)	Risk allele (T) Freq
Discovery cohort	Controls	123	31	1	0.11	77	70	8	0.28
	Cases	154	91	21	0.25	78	107	81	0.51
Replication cohort	Controls	58	6	1	0.06	23	29	13	0.42
	Cases	54	8	0	0.06	7	21	34	0.72
Allele frequency cohort	European clade	1523	676	69	0.18	772	1048	448	0.43
	North American clade	2006	232	24	0.06	526	1084	652	0.53

As the significance of the minor locus (*PRKAA2*) was lost after including the North American population, the possible contribution of a population‐specific difference was assessed. Allele frequencies for top SNP AX‐167725746 (chr5:53062339) were compared between the European and North American populations and analyzed separately for cases and controls within each population (Table 2).

### 3.5 Model for Risk Prediction

To estimate the disease‐risk associated with the DCM3 and DCM4 variants, all Dobermanns from the Discovery and Replication cohorts with a known phenotype were combined for analysis (Table 3).

**TABLE 3:** Results of the risk analysis for DCM based on the genotype of genes DCM3, DCM4, and sex.

	Estimate	95% confidence interval
Reference: DCM3 AA, DCM4 AA, Male	0.99^a^	0.70–1.40
DCM3 genotype 1 AG	2.11^b^	1.35–3.34
DCM3 genotype 2 GG	5.39^b^	1.45–35.10
DCM4 genotype 1 AT	1.32^b^	0.88–1.99
DCM4 genotype 2 TT	5.08^b^	2.97–8.96
Female	0.58^b^	0.40–0.84

^a^ Odds in reference: DCM3 genotype 0 (AA), DCM4 genotype 0 (AA), Male.

^b^ Odds Ratio: the odds for DCM is Estimate times as large as the odds for DCM in the reference.

When considering sex and the genotypes for DCM3 and DCM4, using a cutoff value of 0.625 for the risk probability (Youden index), and the area under the curve, the model (AUC: 0.669, 95% CI: 0.624–0.714) achieves a specificity of 0.845 and a sensitivity of 0.464.

**FIGURE 5:**
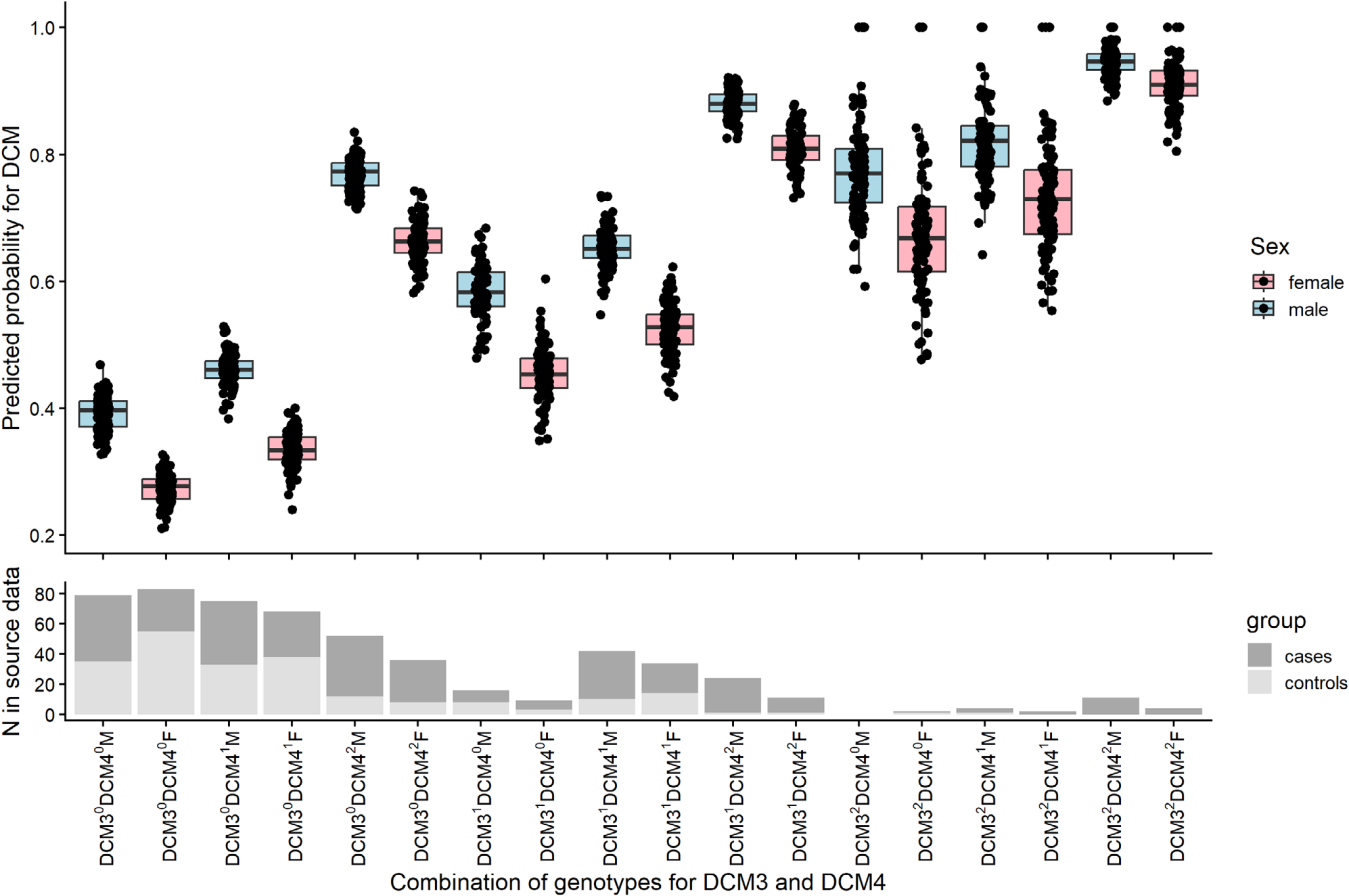
Risk prediction for DCM by combining sex and genotypes for DCM3 and DCM4. Top: Calculated risk prediction for DCM by cross validation of 100 runs when modeling sex with genotypes for DCM3 and DCM4, with 0 = homozygous reference; 1 = heterozygous; 2 = homozygous variant. Bottom: The number of cases and controls used for the risk prediction per combination of risk alleles of DCM3 and DCM4 and sex.

For the DCM3 variant, an additive inheritance pattern was observed. The proportion of affected dogs increased progressively with the number of risk alleles, with heterozygous dogs showing an intermediate risk (Odds Ratio (OR) = 2.11) compared with homozygous reference and homozygous variant genotypes, which showed an increased risk (OR = 5.39). In contrast, the DCM4 variant followed a recessive inheritance model. Dogs homozygous for the risk allele showed a markedly increased prevalence of DCM (OR = 5.08), whereas heterozygous dogs had a slightly increased risk (OR = 1.32) compared to dogs homozygous for the reference allele. Sex‐specific analyses revealed differences in disease risk. Across genotypes, male Dobermanns showed a higher prevalence of DCM than females, indicating an overall increased risk for males. A full overview of the predicted probabilities for DCM in 18 genetic profiles when combining sex, DCM3 and DCM4 is depicted in Figure 5.

### 3.6 Pathogenicity Classification of RNF207 Variant

As genetic testing in domestic animals becomes increasingly accessible, misinterpretation of results can have serious consequences for both individual animals and overall breed health. To support standardized assessment of disease‐associated variants, Animal Variant Classification Guidelines (AVCG) have been developed (Boeykens et al. 2024). The AVCG was applied to assess the pathogenicity of the *RNF207* variant (DCM4; chr5:60111983 G>A; c.1297‐1G>A). Previous studies have evaluated multiple aspects of this splice‐site variant, which results in the loss of the first nine bases of exon 13 and the consequent deletion of three amino acids. These include computational RNA analyses (AVCG criterion PP3; predicted impact on the coiled‐coil domain) and functional association analyses (AVCG criterion PS3, moderate evidence; altered protein expression in cardiac tissue) (Niskanen et al. 2023). Our data further demonstrate a significantly higher allele frequency in affected dogs, consistent with AVCG criterion PS4. Overall, according to the AVCG, the available evidence supports classification of the *RNF207* variant as likely pathogenic.

## 4 Discussion

Different genetic risk alleles for DCM have been identified in the Dobermann breed across various geographical regions. Understanding their global distribution is crucial for breed health and the development of effective screening strategies. Recently, a functional variant in the *RNF207* gene and an association signal at the *PRKAA2* locus have been identified in European Dobermanns (Niskanen et al. 2023). The *RNF207* gene encodes a RING‐finger protein with a proposed role in modulating cardiac electrical activity and hypertrophy. In humans, dysfunctional RNF207 protein activity has been linked to the pathophysiology of long‐QT syndrome (Roder et al. 2014). *PRKAA2* encodes the α2 catalytic subunit of AMP‐activated protein kinase, a key regulator of cellular energy homeostasis. To assess the broader relevance of the *RNF207* and *PRKAA2* genes, our study aimed to evaluate these risk variants in the North American Dobermann population.

Our findings strongly support that the *RNF207* variant functions as a breed‐wide genetic risk factor for DCM. Although our PCA identified a clear genetic separation between the North American and European subpopulations, the association signal at the *RNF207* locus was present in both subpopulations. In our GWAS, the new top SNP at position chr5:60168651 positions even closer to the *RNF207* variant (chr5:60111983G>A, c.1297‐1G>A) than the previously reported top SNP (chr5:60531090). Further, in the North American Dobermanns with unknown DCM status, our large Allele frequency cohort, the associated risk allele near *RNF207* was also found at a high frequency of 0.53. This aligns with the high global prevalence of DCM reported in Dobermanns. The common presence of the *RNF207* risk allele in our cohort, therefore, supports its potential relevance for Dobermanns worldwide. Also, a loss of the association signal near the *PRKAA2* locus was observed. The absence of a clear candidate genomic variant underlying *PRKAA2* splicing abnormalities, as reported previously, may contribute to the weak association signal observed in this region in the present study. In the European population, allelic variation, although low, is present, which may underlie the clearer signal observed. Although previous studies in the North American Dobermann population identified mutations in *PDK4* and *TTN* associated with DCM (Meurs et al. 2012, 2020), we did not observe associations at the loci overlapping these genes in our cohort of North American Dobermanns. This lack of replication might be attributed to the fact that we did not genotype the reported variants or differences in study design, such as cohort size and genomic array density. Alternatively, it remains possible that the originally reported associations do not reflect true underlying effects. For instance, if they arose through statistical chance given the sample sizes involved. We cannot distinguish between these explanations based on the present data. Similar findings were reported in a study on DCM in a cohort of Dobermanns from the United Kingdom. Here, 74 Dobermanns were genotyped at the *RNF207*, *PDK4*, and *TTN* loci, and a clear association with DCM was only found for *RNF207* (Dutton et al. 2025), which in turn was in line with the findings in the original European Discovery cohort (Niskanen et al. 2023).

Cross‐validation of the multivariate logistic regression model, including the number of DCM3 and DCM4 risk alleles and sex, indicated that DCM3 showed a modest effect, while DCM4 exhibited the strongest individual effect on disease status. Given the late onset of DCM in Dobermanns, some control dogs, particularly those carrying the homozygous risk genotype, may have been classified before disease manifestation. This potential misclassification could lead to an underestimation of the true effect size of the *RNF207* variant. Because the training set used a balanced case:control ratio, the model's predicted probabilities should not be interpreted as precise estimates of absolute DCM risk in the general Dobermann population; however, given the high reported prevalence of DCM in this breed (up to 58.2% (Wess et al. 2010)) the 1:1 training ratio is reasonably close to the underlying population base rate, so these probabilities likely approximate absolute risk more closely than would be expected under a more typical case–control imbalance correction. When both loci were included simultaneously, the combined model achieved an AUC of 0.669 (95% CI 0.624–0.714), consistent with an additive contribution of the two loci to predictive accuracy. The model showed high specificity but relatively low sensitivity for predicting DCM. The high specificity indicates that non‐DCM‐affected dogs without the risk genotypes were correctly classified as unaffected in most cases, suggesting that these variants can reliably identify dogs with a lower genetic risk. However, the low sensitivity indicates that many affected dogs do not carry these variants, consistent with the complex genetic architecture of DCM in Dobermanns. From a breeding perspective, DCM3 and DCM4 should therefore be considered supportive risk markers rather than definitive predictors of disease status. Excluding all carriers from breeding could unnecessarily reduce genetic diversity without substantially lowering disease prevalence in the population. We recommend that genetic test results be used alongside, rather than in place of, regular clinical screening when making breeding and monitoring decisions, with dogs carrying risk genotypes prioritized for more frequent or earlier clinical screening rather than automatic exclusion from breeding.

Genotype imputation was performed to increase marker density and to harmonize datasets generated on different SNP array platforms, enabling combined analysis across cohorts. In within‐breed canine populations, where long linkage disequilibrium blocks are common, increased variant density improves tagging of potential causal variants and enhances the resolution of association signals. Moreover, imputation facilitates direct comparison of allele frequencies and haplotypes across subpopulations, enabling evaluation of population‐specific effects and improving the robustness of cross‐population association analyses.

A limitation of this study is the phenotyping of the Replication cohort, which was based on owner‐reported questionnaire data rather than standardized clinical evaluation, and may therefore be subject to misclassification. Although the questions were concise and the dog owners' responses clear, objective clinical data were lacking. Echocardiographic evaluations and Holter monitor results were available for only a small number of dogs; therefore, we relied largely on the owners' responses. This may have impacted the reliability of phenotype classification. Furthermore, misclassification may have occurred in cases of preclinical DCM that remained unrecognized due to the absence of clinical signs at the time of reporting. We aimed to address this by applying a relatively high age threshold for controls. The questionnaire did not allow for multiple selections for cause of death, but instead focused on the primary cause. This could lead to missing additional cases, which would currently be defined as unknown in our allele frequency cohort. In addition, we did not perform conditional analyses on the RNF207 splice‐site variant (chr5:60111983 G>A) in the merged cohort, nor did we fine‐map the chr5 locus. However, within‐breed canine populations, where long linkage disequilibrium blocks are common, increased variant density improves tagging of potential causal variants and enhances the resolution of association signals.

## 5 Conclusion

This study points toward a significant role for *RNF207* (DCM4) in the genetic basis of DCM across multiple Dobermann populations. Beyond its relevance for canine health, the finding establishes the dog as a valuable spontaneous model for RNF207‐driven DCM, creating opportunities to study disease biology and translational cardiac mechanisms in a naturally occurring setting. In contrast, breeding decisions based on potentially nonpathogenic genetic variants, such as DCM1 and DCM2, may have serious adverse consequences for a further decrease in the genetic diversity of the Dobermann breed. Hence, there is a clear need to re‐evaluate the genetic markers currently in use. Future breeding strategies should be applied cautiously and should prioritize robustly validated markers to improve the long‐term health of the Dobermann breed.

## Author Contributions

**Alma H. Hulsman:** writing – original draft, methodology. **Gwen Flokstra:** data curation, formal analysis, visualization, methodology, writing – original draft. **Christina Kijan:** data curation, methodology, writing – review and editing. **Marilijn van Rumpt:** data curation, formal analysis, writing – original draft. **Johannes C. M. Vernooij:** formal analysis, visualization, writing – original draft. **Thomas C. Nelson:** data curation, writing – review and editing. **Sophie Liu:** data curation, writing – review and editing. **Lieke Vree:** formal analysis, methodology, writing – review and editing. **Magdalena Harakalova:** writing – review and editing. **Ingrid Ljungvall:** writing – review and editing. **Jens Haggstrom:** writing – review and editing. **Gerhard Wess:** writing – review and editing. **Hille Fieten:** conceptualization, writing – review and editing. **Marjo K. Hytönen:** data curation, writing – review and editing. **Hannes Lohi:** writing – review and editing. **Frank G. van Steenbeek:** conceptualization, formal analysis, supervision, visualization, writing – original draft.

## Conflicts of Interest

T.N. is employed by Embark, a commercial provider of genetic testing services; however, this affiliation did not influence the design, conduct, analysis, interpretation, or conclusions of the study.

## Supporting information


**Data S1:** Supporting Information.

**Figure S1:** Sample flow diagram. The diagram shows the cohort assembly, quality‐control exclusions, and assignment of the Discovery and Replication cohorts to European and North American subpopulations. The Allele frequency cohort was used solely for population‐level allele frequency estimation and was not included in downstream case–control or risk‐prediction analyses.

**Figure S2:** Principal component analysis based on the Allele frequency cohort. Each point represents an individual, colored by phenotype status. The dashed line marks the approximate boundary between the North American and European population.

## Data Availability

The data that support the findings of this study are openly available in DataverseNL at https://doi.org/10.34894/QDKTS7, reference number 10.34894/QDKTS7.
