# Supplementary material for: A Large‐Scale Cross‐Population Evaluation of Genetic Risk Variants for Dilated Cardiomyopathy in Dobermanns
Source: Anim Genet. 2026 Sep 9;57(5):e70206. doi: 10.1002/age.70206 (PMC13558464; doi:10.1002/age.70206)
Supplement: Supplementary file 1 [file AGE-57-0-s002.docx]

**Supplementary Data: Embark Dobermann Survey**

The Qualtrics questionnaire shown below was sent to owners of Dobermanns after they had completed Embark genetic testing. Only the items relevant to the current study are included here. As the questionnaire was interactive, the arrows indicate the response pathways followed by participants depending on their answers.

**Block 1 – Intro**

Embark is working with Utrecht University to identify potential genetic causes underlying certain health conditions among Doberman Pinschers. By continuing with this survey about [name]'s health, you agree that Embark may share your responses with Utrecht University. All responses are confidential and will be used for research purposes only. Thank you for contributing to this important research.

Q1. How would you rate [name]'s health, currently?

- Excellent 🡪 Block 2, 4-7
- Good 🡪 Block 2, 4-7
- Fair 🡪 Block 2, 4-7
- Poor 🡪 Block 2, 4-7
- Deceased 🡪 We're so sorry to hear this. Everyone at Embark knows how important dogs are to our lives and how hard it is to lose them. [Name] and you will always be a part of the Embark pack.

Q2. Are you comfortable answering some additional questions about [name]'s health for research purposes?

- Yes 🡪 Block 2, 3 and 7
- No 🡪 Block 7

**Block 2 – Date of Birth**

Q3. Please enter [name]'s birthdate (known or estimated).

- Month/Day/Year

**Block 3 – Deceased**

Q4. When did [name] pass away?

- Month/Day/Year

Q5. Could you tell us the manner of [name]’s passing?

- Euthanasia 🡪 Q6
- Natural death (including disease or complications from medical procedure / intervention) 🡪Q7
- Traumatic injury / accident (e.g., vehicle accident)
- Prefer not to say
- I don't know

Q6. Could you tell us the primary cause that led to [name]’s euthanasia?

- Dilated cardiomyopathy
- Doberman hepatitis
- As a result of an accident / trauma
- Behavioral problems (e.g., aggression)
- Cancer
- Other chronic illness (e.g., heart or kidney disease, immune-mediated disease, etc.)
- Complications as a result of a medical procedure (e.g., surgery)
- Infectious disease or parasites (e.g., parvovirus, distemper, Lyme disease, heartworm, etc.)
- Old age / quality of life concerns (e.g., mobility issues, incontinence, pain, etc.)
- Other sudden illness (e.g., bloat, heart or kidney failure, pancreatitis, etc.)
- Prefer not to say
- I don't know
- Other - please specify:

Q7. Could you tell us the primary cause that led to [name]’s passing?

- Dilated cardiomyopathy
- Doberman hepatitis
- Cancer
- Other chronic illness (e.g., heart or kidney disease, immune-mediated disease, etc.)
- Complications as a result of a medical procedure (e.g., surgery)
- Infectious disease or parasites (e.g., parvovirus, distemper, Lyme disease, heartworm, etc.)
- Old age / quality of life concerns (e.g., mobility issues, incontinence, pain, etc.)
- Other sudden illness (e.g., bloat, heart or kidney failure, pancreatitis, etc.)
- Prefer not to say
- I don't know
- Other - please specify:

**Block 4 – Intact status**

Q8. Has [name] been spayed or neutered?

- Yes
- No 🡪 Q9
- I don’t know

Q9. Are you planning to have [name] spayed or neutered?

- Yes
- No
- I don’t know

**Block 6 – DCM**

*The following questions ask about dilated cardiomyopathy.*

Q16. Has [name] been diagnosed with dilated cardiomyopathy (DCM) by a veterinarian?

- Yes 🡪 Q17, Q18
- No
- I don’t know

Q17. Approximately how old was [name] when diagnosed with DCM?

- …

Q18. How was [name] diagnosed with DCM? *Select all that apply.*

- Echocardiogram/ultrasound
- Holter monitoring
- Chest x-rays
- None
- Other, please describe: …

**Block 7 – Additional Info**

Is there anything else you'd like to tell us about [name]'s health?

- **…**

Thank you for completing this survey. Researchers at Utrecht University may want to reach out to you to gather more information about [name]'s health, which could involve direct email contact and a more in-depth questionnaire provided by the research team.

Do you grant Utrecht University permission to contact you directly

- Yes
- No

**Supplementary Data: Details of the model for risk prediction**

Area under the curve: 0.6687

95% CI: 0.6235-0.7138 (DeLong)

Youden Threshold : 0.6251741

=== Confusion matrix: prediction x status

                    DCM-(prop)   DCM+(prop)

predict low      0.845  (spec)    0.536

predict high      0.155                   0.464 (sens)

95%BI sensitivity: 0.41 – 0.52

95%BI specificity: 0.80 - 0.89
